# Plant and metagenomic DNA extraction of mucilaginous seeds

**DOI:** 10.1016/j.mex.2014.09.005

**Published:** 2014-10-13

**Authors:** Simone N.M. Ramos, Marcela M. Salazar, Gonçalo A.G. Pereira, Priscilla Efraim

**Affiliations:** a Department of Food Technology, School of Food Engineering, University of Campinas, Brazil; b Laboratory of Genomic and Expression, Institute of Biology, University of Campinas, Brazil

**Keywords:** Plant, Seeds, Cupuassu, DNA extraction, PCR, Metagenomic

## Abstract

The pulp surrounding the seeds of some fruits is rich in mucilage, carbohydrates, etc. Some seeds are rich in proteins and polyphenols. Fruit seeds, like cacao (*Theobroma cacao*) and cupuassu (*Theobroma grandiflorum*), are subjected to fermentation to develop flavor. During fermentation, ethanol is produced [2–6]. All of these compounds are considered as interfering substances that hinder the DNA extraction [4–8]. Protocols commonly used in the DNA extraction in samples of plant origin were used, but without success. Thus, a protocol for DNA samples under different conditions that can be used for similar samples was developed and applied with success. The protocol initially described for RNA samples by Zeng et al. [9] and with changes proposed by Provost et al. [5] was adapted for extracting DNA samples from those described. However, several modifications have been proposed:

• Samples were initially washed with petroleum ether for fat phase removal.
• RNAse was added to the extraction buffer, while spermidin was removed.
• Additional steps of extraction with 5 M NaCl, saturated NaCl and CTAB (10%) were included and precipitation was carried out with isopropanol, followed by washing with ethanol.

## Graphical abstract

Stages of sample treatment: (a) seeds in fermentation boxes; (b) frozen cupuassu beans; (c) maceration of beans with liquid nitrogen; (d) fat extraction under stirring with petroleum ether; and (e) initial step of DNA extraction process.

## Background information

The pulp surrounding the seeds of some fruits is rich in mucilage, carbohydrates, proteins and polyphenols. Fruit seeds, like cacao (Theobroma cacao) and cupuassu (Theobroma grandiflorum), are subjected to fermentation to develop flavor giving products like chocolate and “cupulate”, respectively. During fermentation, because of the intense biochemical reactions that occur in the event, there is the formation and release of ethanol and phenolic compounds such as flavonoids [1,2,3,6]. Samples of plant origin with high concentration of mucilage have a difficult DNA extraction. Besides the mucilage, there are other types of interferences, such as carbohydrates, proteins and phenolic compounds that hamper the DNA extraction process. To avoid this inconvenience, it is necessary to test several types of protocols aiming to obtain genomic DNA, and the relationship between nucleic acid and protein (*A*_260_/*A*_280_), nucleic acid and carbohydrates (*A*_260_/*A*_230_) must be within acceptable limits [4,8].

Different protocols for DNA extraction, including the use of commercial Kits were tested with no success for the DNA extraction of fermented cupuassu seeds (beans). The protocol developed in this work combines different methodologies to decrease the high carbohydrate content that usually accompanies the DNA extracted from mucilaginous samples.

Cupuassu seeds have a high concentration of phenolic compounds, mainly in unfermented seeds [6]. During fermentation, because of the intense biochemical reactions that occur in the event, there is the formation and release of phenolic compounds such as flavonoids, but the concentration of these substances decreases throughout the process and during the drying of beans [1]. Although there is a decrease in the concentration of polyphenols during fermentation, the remaining compounds, mainly flavonoids, may interfere in the DNA extraction procedure of the samples [7]. The aim of this study was to standardize a protocol for total (plant and metagenomic) DNA extraction of fermented cupuassu seeds, since protocols commonly used for DNA extraction from samples of plant origin showed no satisfactory results in this work. Thus, the protocol initially described for RNA samples by Zeng et al. [9] and with changes proposed by Provost et al. [5] was adapted for extracting DNA samples from those described.

## Method details

### Preparation of material

Seeds of cupuassu (*Theobroma grandiflorum* Schum) freshly harvested were used for the fermentation step. The seeds were accommodated in fermentation boxes of polystyrene with capacity of 24 L. The boxes had holes of 2 cm in diameter at the bottom and sides for the flow of the liquefied pulp from fermentation. Chopped banana leaves were mixed to the fermentation mass, which act as a natural inoculum, and then the mass was covered with banana leaves. The fermentation process had duration between 68 and 116 h. Approximately 12 g of fermented seeds were collected in triplicate. The procedure of DNA extraction was developed as follows.

### Maceration and fat removal

**Fig. 1:**
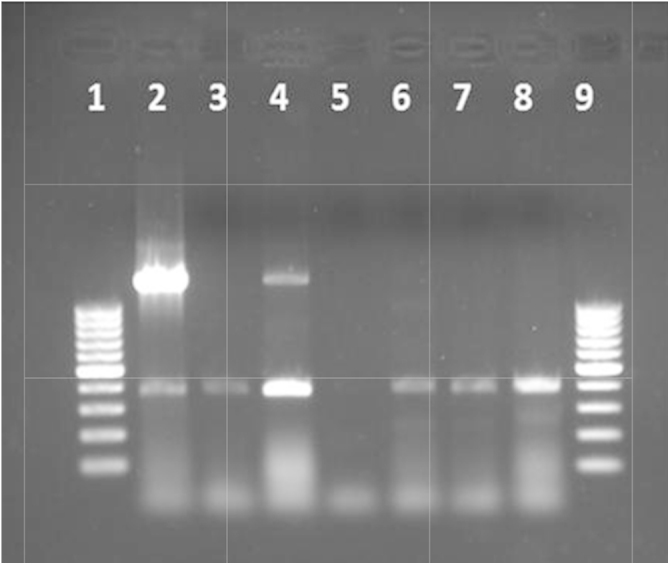
Agarose gel (2%) showing amplification with 16S primers of the DNA extracted from fermented samples. 2–4: samples in the first day of fermentation; 5: non-fermented sample; 6–8: samples in the last day of fermentation; 1 and 9: 100 bp ladder LifeTechnologies.

(1) Pulverize, using mortar and pestle, of 12 g of fermented seeds in liquid nitrogen (Fig. 1b and c).
(2) Wash samples (1 g of pulverized sample) with 9 mL of petroleum ether (in 15 mL tubes) under agitation for 5 min (three times) for fat phase removal (Fig. 1d).
(3) Keep tubes open in a fume hood for total evaporation of petroleum ether (∼5 min).
(4) Transfer approximately 300 mg of defatted samples to a 2 mL tube and freeze them in liquid nitrogen.
(5) For finer material, pulverize again of samples in a MiniBeadBeater (Biospec Products) for 1 min and a half with ∼9 glass beads (1.0 mm diameter).

### DNA extraction

(6) Add 1 mL of pre-heated extraction buffer (2% CTAB, 2% polyvinylpyrrolidone, 100 mM Tris–HCL (pH 8.0), 25 mM EDTA, 2.0 M NaCl, 10 mg RNAse, 10% β-mercaptoethanol) to the frozen samples and keep them for 30 min in a dry bath (65 °C), vortexing every 5 min.
(7) Wait for samples to reach room temperature and add 300 μL of saturated NaCl. Centrifuge at 10,000 × *g* at 4 °C for 10 min.
(8) Transfer the supernatant (Fig. 1e) to another 2 mL tube, add 160 μL of 5 M NaCl and homogenize.
(9) Add 600 μL of CIA (chloroform and isoamyl alcohol 24:1) and centrifuge at 10,000 × *g* at 4 °C for 10 min.
(10) Transfer the superior phase to another 2 mL tube. Add 50 μL of CTAB (10%) solution with 1.4 M NaCl solution and vortex. Add 600 μL of CIA, homogenize by inversion and centrifuge.
(11) For the superior phase, repeat the CIA extraction.
(12) Transfer the resulting superior aqueous phase to a new 1.5 mL tube and add cold isopropanol (fill the tube) for DNA precipitation (−20 °C for 12 h).
(13) Centrifuge samples (10,000 × *g*, at 4 °C for 30 min) and wash the resulting pellet twice with 400 μL of 70% ethanol for 30 min and once with absolute ethanol for 3 min.
(14) Dry pellets in a dry-bath at 70 °C for 5 min and resuspend in 40 μL of DNAse-free water.

### Purification

Eventually, a co-extraction of RNA and PCR inhibitors can occurs [7]. In order to avoid future problems with the genomic analysis, perform the purification steps using the DNeasy Plant Minikit (Qiagen).

### DNA yields, quality and PCR conditions

Verify DNA concentration and quality with a Nanodrop 2000 instrument (Thermo Scientific). In this work results showed that the concentration in ng of all samples were within the optimal conditions (low concentration of proteins and great amount of DNA extracted), varying between samples. Perform a PCR with universal *16S* and *ITS* primers and with GoTaq polymerase (Promega), as follows: 1× GoTaq Buffer, 10 mM MgCl_2_, 40 μM dNTPs, 5 mM primers, 10 μ GoTaq, 100 ng DNA. Carry out PCR with an initial denaturation step at 94 °C, 2 min; then, 30 cycles of denaturation (94 °C, 40 s), annealing (50 °C, 30 s) and elongation (72 °C, 1 and a half minutes) and a final step of elongation for 4 min. The results can show that despite small amounts of carbohydrates still being observed in DNA samples, they did not impair the amplification of bacterial DNA content present in the total DNA of fermented seeds.

Different protocols for DNA extraction, including the use of commercial Kits were tested with no success for the DNA extraction of fermented cupuassu seeds (beans). The protocol developed in this work combines different methodologies to decrease the high carbohydrate content that usually accompanies the DNA extracted from mucilaginous samples.

The quality of samples and their concentrations were verified to attest the efficiency of the DNA extraction method. Results are available in Table 1 and Fig. 1.

## Additional information

The results showed that the concentration in ng of all samples were within the optimal conditions (low concentration of proteins and high amount of DNA extracted), varying between samples. To confirm the quality of the DNA samples, a PCR was performed using 16S primers for samples at the third day of fermentation (with 15% of pulp). These results showed that despite small amounts of carbohydrates still being observed in DNA samples, they did not impair the amplification of bacterial DNA content present in the total DNA of fermented seeds.

The protocol developed in this work was successfully applied for the DNA extraction of fermented *T. grandiflorum* seeds, thus confirming that the protocol can be used for the DNA extraction of similar samples. The age and growth conditions of the plant material influence the isolation efficiency of high-quality DNA. Currently, it permits further genomic analyses.

## Figures and Tables

**Table 1:** Concentration and quality of DNA extracted from mucilage samples of fermented cupuassu seeds.

Sample (code)	DNA amount (ng μL^−1^)	*A*_260_/*A*_280_	*A*_260_/*A*_230_
CUP03	104.5	2.13	2.18
CUP06	74.8	2.19	1.90
CUP15	86.5	2.13	2.01
CUP16	222.0	2.13	2.23
CUP18	51.1	2.04	1.07
CUP19	136.4	2.13	2.12
CUP22	70.5	2.13	2.54
CUP28	70.5	2.17	1.85
CUP39	71.4	2.11	1.87
CUP40	64.0	2.09	2.10
CUP41	105.9	2.12	2.18
CUP44	116.3	2.03	1.66
CUP45	96.9	2.12	2.05
CUP47	311.8	2.11	1.96
CUP51	93.7	2.12	2.19
CUP54	141.4	2.12	2.26
CUP58	61.4	2.10	2.23
CUP59	161.5	2.10	2.13
CUP62	67.9	2.14	2.27
CUP63	64.6	1.81	0.78
CUP64	78.9	2.10	2.14
CUP65	64.3	2.11	2.45
CUP66	150.2	1.93	0.94
CUP71	74.6	2.14	2.24
CUP76	51.4	2.08	1.78
CUP78	63.6	2.04	1.28

## References

[1] Beckett S. (2009). Industrial Chocolate Manufacture and Use.

[2] Carvalho A.V., García N.H.P., Wada J.K.A. (2005). Caracterização físico-química e curvas de solubilidade proteica de sementes, amêndoas fermentadas e torradas de cupuaçu (*Theobroma grandiflorum* Schum). Braz. J. Food Technol..

[3] Cohen K.d.O., Sousa M.d., Jackix M. (2009). Produto Alimentício Elaborado com Sementes de Cupuaçu e de Cacau.

[4] Kundu A. (2001). A simple ethanol wash of the tissue homogenates recovers high-quality genomic DNA from Corchorus species characterized by highly acidic and proteinaceous mucilages. Electron. J. Biotechnol..

[5] Provost G.L., Herrera R., Paiva J.A., Chaumeil P., Salin F., Plomion C. (2007). A micromethod for high throughput RNA extraction in forest trees. Biol. Res..

[6] Pugliese A.G. (2013). Flavonoids, proanthocyanidins, vitamin C, and antioxidant activity of *Theobroma grandiflorum* (cupuassu) pulp and seeds. J. Agric. Food Chem..

[7] Schrader C. (2012). PCR inhibitors – occurrence, properties and removal. J. Appl. Microbiol..

[8] Sharma P.K., Capalash N., Kaur J. (2007). An improved method for single step purification of metagenomic DNA. Mol. Biotechnol..

[9] Zeng Y., Yang T. (2002). RNA isolation from highly viscous samples rich in polyphenols and polysaccharides. Plant Mol. Biol. Rep..

